# Male involvement in sexual and reproductive health in the Mendi district, Southern Highlands province of Papua New Guinea: a descriptive study

**DOI:** 10.1186/1742-4755-10-46

**Published:** 2013-09-10

**Authors:** Sally Kura, John Vince, Paul Crouch-Chivers

**Affiliations:** 1 School of Medicine and Health Sciences, University of Papua New Guinea, PO Box 5255, Boroko, Port Moresby, Papua New Guinea

**Keywords:** Married men, Knowledge, Male involvement, Wives’ reproductive health, Sexual behaviour

## Abstract

**Background:**

Lack of male involvement and support for sexual and reproductive health services is seen by many Papua New Guinean women as a barrier to accessing services. Poor utilization of services by both men and women is reflected in high maternal mortality and high rates of HIV/AIDS and sexually transmitted infections in the Southern Highlands Province. It is therefore important to understand the type of services provided, men’s perceptions of these services and the Health Sector’s capacity to involve men in its programs.

**Methods:**

Information from interviews of married men, officers in charge of health facilities, and information from a focus group discussion with village leaders was collected to assess possible constraints to reproductive and sexual health care delivery.

**Results:**

Although many men had heard about antenatal care, supervised births, family planning and sexually transmitted infections including, HIV/AIDS, many were unaware of their importance and of the types of services provided to address these issues. There was a very strong association between men’s literacy and their knowledge of Sexual and Reproductive Health (SRH) issues, their discussion of these issues with their wives and their wives’ utilisation of sexual and reproductive health services. Some men considered SRH services to be important but gave priority to social obligations. Although men made most decisions for sexual and reproductive issues, pregnancy, child birth and rearing of children were regarded as women’s responsibilities. Knowledge of HIV/AIDS appeared to have changed sexual behaviour in some men. Services for men in this rural setting were inadequate and service providers lacked the capacity to involve men in reproductive health issues.

**Conclusion:**

Poor knowledge, socio-cultural factors and inadequate and inappropriate services for men hampered utilization of services and impaired support for their wives’ service utilization. Programmatic and policy initiatives should focus on improving service delivery to accommodate men in sexual and reproductive health.

## Background

Whilst global recognition of the importance of male involvement in sexual and reproductive health (SRH) emanated from the 1994 International Conference on Population and Development held in Cairo [1], it is a relatively new concept in Papua New Guinea (PNG). In PNG women have been the principal targets of sexual and reproductive health services over the past decades and men’s participation has been minimal. In rural areas, where more than 80% of the population live, the availability of quality sexual and reproductive health services for men is inadequate. Service utilization for SRH services has been low. National annual average figures for 2006–2010 for family planning (couple protection rate per year) were 83/1000 women, antenatal coverage 67% and supervised deliveries less than 50% [2]. This is reflected in very high maternal (733/100,000 live births^a^) infant (56.7/1000 live births) and neonatal (29.1/1000 live births) mortality rates [3].The highest maternal death rates have been reported from the highlands region. The majority of maternal deaths are preventable with access to services.

A preliminary, smaller and unpublished study in the Southern Highlands by the first author strongly suggested that lack of male involvement and support for utilization of safe motherhood services is seen by women as a barrier to accessing services. (Project for Diploma of Public Health, University of Papua New Guinea 2010). The Southern Highlands Province has experienced an increase in sexually transmitted infections (STIs), including HIV/AIDS, suggesting poor male involvement in HIV/AIDS and STI prevention. HIV prevalence in PNG is currently estimated at 0.92% with a preponderance of females [4]. Heterosexual sex is by far the commonest mode of transmission [5]. HIV and STIs are more easily transmitted from men to women than women to men [6,7]. Women in PNG are at risk of contracting STI and HIV because of cultural and social norms by which wives cannot decline sexual intercourse with their husbands nor can they insist that their partners use condoms. Whilst there are differences between some aspects of HIV/AIDS in different countries it is almost certain that in PNG as in Sub-Saharan Africa the majority of HIV positive women have been infected by their stable partners [8]. Whilst pregnancy child birth and the rearing of children as well as domestic chores are regarded as women’s responsibilities husbands play a pivotal role in decision making within the home and obtaining their involvement in and support for interventions to improve sexual and reproductive health for themselves and their wives is critical [9,10].

A holistic approach is required. If men are involved and supported as equal partners better outcomes in sexual and reproductive health indicators, such as contraception acceptance, safer sexual behaviours, use of reproductive health services and reduction in SRH related morbidity and mortality, can be expected [11]. Results from Ghanaian [12] and Indonesian [13] studies demonstrate that a program that focuses on improving men’s knowledge of SRH for themselves and their partners is likely to be more effective than one that targets women. Involving men in SRH requires in-depth knowledge and understanding of men’s sexual and reproductive health perspectives [14,15]. This study aimed to explore and assess the knowledge, attitude and role of men from the Mendi district of Southern Highlands province in regards to antenatal care, supervised birth, family planning and prevention of HIV/AIDS/STI and to assess the service providers’ capacity to involve men in its programmes.

## Methods

In this prospective descriptive study data collection came from three sources; individual interviews with married men, interviews with health workers and focus group discussions with village leaders.

The study population was married men between the ages of 15–44 currently residing in a household with their wife/wives and who had fathered at least one child. (In this context a wife is defined as a woman living with a man in a non-casual relationship which is accepted within the local culture).

Council wards were selected by the lucky pick technique [16]. The names of all the council wards in each of the three Local Level Government areas were written on individual papers which were folded and put into the respective area box. Following mixing ten wards were selected from each box [16]. For each of the 30 selected wards, a list of men conforming to the selection criteria was provided by the local health workers and 7 men from each ward were selected using the same technique. In-depth structured interviews of the 210 selected men, using a pretested questionnaire were conducted by trained research assistants.

Data regarding service factors were collected from officers in charge of six district health facilities using semi-structured, self-administered questionaries to capture information on the types of SRH services offered at each health facility, whether those services were targeted at men or women, and the health workers’ views and experiences of working with men. Purposive sampling was used to select Officers in Charge (OICs) known to have broad knowledge and experience in all aspects of health care delivery in their establishment. The questionnaire was distributed to the participants and the researcher explained the instruments and clarified any misunderstandings while the respondents answered the questions.

Two focus group discussions were held with 12 village leaders from Upper Mendi and Lai Valley local level government areas to seek views on specific issues and to validate findings from the individual interviews. Purposive sampling was used in the selection of participants who had different leadership roles but possessed similar social and cultural backgrounds, were well acquainted with the social and health issues in their respective communities and had comfortably engaged in dynamic discussions. A Digital Voice Recorder was used in recording all the discussions. The principal investigator moderated the FGD while one of the research assistants did manual recording.

Quantitative data from individual interviews were processed and analysed using the Epi Info (version 3.5.1) computer program. A descriptive analysis was performed for each variable. Chi-square or Fisher exact test was performed to explore the relationship between selected variables.

Qualitative data from individual interviews, self-administered questionnaires and FGDs were processed and analysed using thematic analysis following the model described by Parahoo [17] and the standard coding procedure outlined by Strauss and Corbin [18]. Open and axial coding strategies were applied in identifying major themes and sub themes that emerged from the interview data. The major themes for each variable were then summarized in master sheets indicating frequency of responses for each category. Data from non-participant observations and informal discussions were summarized and analysed manually. Main themes were identified. The data from the FGDs were transcribed into a note book. All recorded discussions were transcribed into Pidgin and then translated into English. This was done by the principal investigator and research assistants after each discussion. Only notes relevant to the study were transcribed and summarized in themes and used for examining relationships with other variables. Issues felt to be important which surfaced in the discussion surrounding each theme were identified and written in narrative form.

Ethical Clearance to conduct this study was obtained from the University of Papua New Guinea (UPNG) School of Medicine and Health Sciences, Research Committee. Informed consent was obtained from study participants, who were assured of confidentiality which was ensured by the use of anonymous questionnaires. Verbal consent was also sought from the respective community leaders prior to the focus group discussions.

## Results

### Study participants

The characteristics of the 222 study participants are outlined in Table 1. The age range was from 21–44 with a mean of 36 years. One third of the respondents did not complete primary education. Many who dropped out from school before reaching third grade did not know how to read and write. The majority of uneducated men were from the remoter areas of the district and did not have access to basic services. Thirty five men were polygamous.

**Table 1:** Background characteristics of study participants

**Characteristic**		**No**	**(%)**
Age	15–25	5	(2)
	26–35	94	(42)
	36-45	123	(55)
Education	None	47	(21)
	Primary	109	(49)
	High school/Secondary	66	(31)
Literate (Stated ability to read and write)		131	(59)
Occupation	Subsistence farmer	162	(73)
	Casual employee	22	(10)
	Village leader	17	(8)
	Church worker	13	(6)
	Public servant	8	(4)
Religion	Roman catholic	65	(29)
	Protestant	157	(71)
Number of Wives	One	187	(84)
	Two	27	(12)
	Three	8	(4)
Number of children	One to two	79	(36)
	Three to four	77	(35)
	Five to six	42	(19)
	Seven to ten	24	(11)

### Antenatal care (ANC) and childbirth

Men’s knowledge of and involvement in the antenatal care of their wives and at the time of delivery is outlined in Table 2.

**Table 2:** Men’s knowledge of and involvement in wive’s antenatal care and delivery

**Parameter**		**n = 210**	**(100%)**	**Details**			
**ANTENATAL CARE**							
Heard of antenatal clinic		140	(67)	Considered antenatal care important		117	(84)
				Knowledge of screening tests	HIV	72	(51)
					VDRL	52	(37)
					Hb	9	(6)
				Knowledge of tetanus vaccine		10	(7)
Wife attended AN clinic		123	(59)	Husband supported wife		37	(30)
				Reasons not supported			
					Poor knowledge	31	(36)
					Facility access	30	(35)
					Culture/gender norms	25	(29)
Wife not attended		87	(41)	Reasons for non-attendance			
					Poor Knowledge	3	(84)
					Facility access	8	(9)
					Male health staff	6	(7)
Spousal discussion	Yes	69	(33)	Reason for no discussion			
	No	141	(67)		Cultural norms	76	(54)
					Poor knowledge	65	(46)
**DELIVERY**							
Heard of supervised births		137	(65)	Importance	Safe delivery	55	(40)
					Avoid complications	14	(10)
					Don’t know	68	(50)
Who decides on place of delivery?							
	Wife	102	(49)				
	Husband	15	(7)				
	Both	83	(40)				
	Health worker	10	(5)				
Last delivery in village		124	(59)	Reasons for village birth			
					Poor knowledge	83	(67)
					Safe village birth	16	(13)
					Male staff at facility	15	(12)
					Distance	10	(8)
	Health facility	86	(41)	Husband provided support	Yes	62	(72)
					No	24	(28)
				Reasons for no support			
					Lack of knowledge	12	(50)
					Cultural norms	9	(38)
					Lack of resources	3	(13)
Spousal discussion	Yes	106	(51)	Reason for no discussion			
	No	104	(50)		Cultural norms	54	(52)
					Poor knowledge	50	(48)

One third of the respondents were unaware of the risk factors associated with pregnancy and the importance of antenatal care. Only 39% of the respondents said that the health of mother and the unborn baby were being checked regularly. The majority viewed ANC as an important service but did not fully support their wives in utilising it. The priority for many men in terms of time and financial matters was given to social obligations such as marriage arrangements and compensation. Committing time and money to social obligations was important in gaining respect and recognition in the community.

Some men leave home and go away when their wife is pregnant to avoid sex which they believe will destroy the baby. Those that never supported their wives viewed pregnancy and childbirth as women’s responsibility and were quite confident of women taking care of themselves.

“*Pregnancy and rearing of children are women’s affair therefore most young men are only interested in making babies*.”(Male 32 from FGD).

“*Men don’t look after their pregnant wives; they think that when their wife dies they will always marry another woman*.”(Male 40 from FGD).

Awareness and education on safe motherhood initiatives have never been targeted at men. Husbands were only informed when their wife encountered problems associated with pregnancy.

*“Health workers do not inform us men about women’s health; therefore we thought it was not necessary for us to know so we mind our own business.”*(Male 30 FGD).

Relatively few husbands supported their wives, mostly in terms of providing encouragement and reminding them of ANC schedules.

Almost two thirds of the participants had heard about supervised births but many were unaware of the type of care provided and the complications associated with labour and delivery. Interaction between men and health workers occurred only when their wives encountered major problems. Women with knowledgeable husbands were more likely to give birth in a health facility than those whose husbands had poor knowledge. Many of those not knowledgeable were from the remoter areas of the District. Many men viewed child birth as an important occasion due to the arrival of a new family member. Some affirmed that their wives comfortably delivered their babies in the village and were being assisted by older women.

*“It’s costly going to the hospital; the older women in the village are experts, they help our wives with the deliveries. All the births of my children occurred in the village.”* (Male 42 FGD).

Men viewed child birth as women’s responsibility; therefore the decision to access services belonged to the women. Participants from focus group stressed that many women delivered in the village because husbands and relatives do not support them in accessing a health facility.

“*In our custom; pregnancy and child birth are women’s job. Therefore, I feel ashamed accompanying my wife to the hospital so I only help her with money. I arrange female relatives to accompany her to the hospital.”* (Male 40 FGD).

### Family Planning (FP)

Men’s knowledge and practice of family planning and related issues is outlined in Table 3.

**Table 3:** Men’s knowledge and practice of family planning

**Parameter**	**n =210**	**(100%)**	**Details**		
Purpose of family planning			Reasons not accessing FP methods		
Spacing births	106	(51)	Poor knowledge	140	(83)
Stops pregnancy permanently	6	(3)	Wanting more children	55	(33)
Don’t know	98	(47)	Religion	31	(19)
			Unavailability of services	31	(19)
			Extramarital affairs	24	(14)
			Sex of health workers	2	(1)
Knowledge of male methods			Reasons for not wanting male methods {n 38)		
Condom	37	(18)	Religion	19	(50)
Vasectomy	0	(0)	Embarrassment	10	(26)
Don’t know	173	(82)	Wife accusing promiscuity	9	(24)
Knowledge of female methods					
Injections	55	(25)			
Pills	43	(21)			
Female condoms	3	(1)			
Tubal ligation	2	(1)			
Intrauterine device	0	(0)			
Don’t know	1 23	(59)			
Current practice			Preferred method in the future		
Abstinence	166	(79)	Male methods	46	(22)
Wife using FP methods	37	(18)	Female methods	38	(18)
Condoms	5	(2)	Don’t know	126	(60)
Ovulation	1	(0.5)			
Withdrawal	1	(0.5)			
Spousal discussion Yes	39	(19)			

Knowledge among the participants on the different types of FP methods was poor. They were unaware of the social, economic and medical benefits of using FP methods. Many thought condoms are used for the prevention of HIV/AIDS but were not aware of their family planning function. Some husbands feared that contraceptives might cause permanent sterility and did not want their wives using contraceptives until they had the desired number of children.

Although individuals in PNG have the right to access contraceptives without their partner’s consent, such access remains a contentious issue, Should a woman request it, health staff may provide a consent form for their husbands to sign. Some of the few who knew about the policy thought it inappropriate for married couples. Participants from a focus group thought such policies are likely to create avenues for increased extra marital relationships.

“*We heard that women while on contraceptives engage themselves in extra marital relationships, therefore; we don’t want our wives to use contraceptives”.* (Male 38 FGD).

A few men provided consent for their wives to access contraceptives and although male methods were available they were not widely used. Consent forms were usually issued to the wife, upon her request, to be taken home to be signed by her husband.

*”A woman had written consent herself, pretended to be written by the husband and presented to the nurse who issued her the contraceptives. Later the husband discovered the pills in the woman’s bag. He bashed her up and summoned the nurse and the wife to the village court*”. (Male 40 FGD).

*“I took some condoms home and my wife accused me of prostitution.”* (Male 35 individual interview).

The condom is considered by many to be associated with promiscuity therefore men felt embarrassed accessing condoms.

It was suggested in a focus group that a male oriented clinic would be ideal for men where they will freely discuss reproductive issues. FP clinics are usually female oriented and men feel embarrassed accessing these services.

Those men not using condoms claimed to have spaced their children by abstaining from sex until their child is able to talk, walk and eat on their own. This practice has been passed through generations and is still being practiced by some men in the district.

### HIV/AIDS

Details of men’s knowledge about HIV/AIDS and their sexual behaviour are shown in Table 4.

**Table 4:** Men’s knowledge of HIV/AIDS and their sexual behaviour

**Parameter**		**No**	**(%)**
Mode of transmission	Sexual intercourse	199	(95)
	Breast milk	122	(58)
	During pregnancy and birth	118	(56)
	Blood transfusion	32	(15)
	Razors	5	(2)
	Needles	4	(2)
	Don’t know	11	(5)
Ways to avoid	One sex partner	144	(69)
	Avoid prostitutes	123	(59)
	Use condom	62	(30)
	Don’t share razor	32	(15)
	Abstain from sex	17	(8)
	Avoid blood transfusion	5	(2)
	Avoid reusable needle	4	(2)
	Don’t know	14	(7)
Knowledge changed sexual behaviour		133	(63)
	One sex partner	105	(79)
	Using condom	14	(11)
	Stopped sex	11	(8)
	Restrict number of partners	3	(2)
Knowledge of voluntary counselling and testing		66	(31)

All those interviewed had heard of HIV/AIDS and the large majority were aware that HIV is transmitted during sexual intercourse with an infected person. Restricting to one sexual partner, avoiding prostitutes and using of condoms were mentioned by many as ways of avoiding HIV/AIDS. More than half the respondents knew that HIV can be transmitted from mother to child during pregnancy, birth and through breast milk. The majority (63.8%) stated that there was no cure for HIV/AIDS but only 14.3% were aware of effective medical treatment available to HIV positive people.

Sixty three per cent claimed that their knowledge of HIV/AIDS had changed their sexual behaviour and they have restricted themselves to one sexual partner. They feared that improper use of condoms might result in contracting the diseases, therefore being faithful to their wives was considered safer than using condoms.

In contrast, 36.7% cited that HIV/AIDS knowledge did not change their sexual behaviour, including those who practiced polygamy. The latter respondents indicated not using condoms when having sex with their multiple wives, stressing difficulty in changing their practices. They thought there was no risk of contracting HIV/AIDS if they only had sex with their multiple wives; they claimed to be fortunate to have multiple wives so they don’t have to go outside of their polygamous relationships looking for sex.

A third of participants indicated having sex with non-cohabiting sexual partners in the previous 12 months and half of them did not use a condom. Many indicated that if they contracted HIV they would not inform their wives or relatives for fear of embarrassment and stigmatization. Most respondents who had previously suffered from STI did not disclose their illness to their wives for fear of argument, retaliation and denial of sex.

### Service factors

The majority of the information regarding service factors was derived from health worker interviews, although some of the men interviewed commented that sexual and reproductive health services, including awareness and education programs, were targeted at women. The services available to men were limited to condom distribution for FP and prevention of HIV/AIDS and only few men accessed them. In the past condoms were placed at private collection points within the health facility for free distribution. However, due to abuse, most facilities now store them out of sight and issue them to clients upon request.

There were no voluntary counselling and treatment services at rural settings and no antenatal HIV screening. Only two facilities performed VDRL tests on pregnant women. HIV screening of antenatal mothers was only done in the Mendi Urban clinic. Patients with simple STI (gonorrhoea & syphilis) were treated at health centres and those suspected of HIV and other STIs were referred to Mendi General Hospital for further diagnosis and treatment.

Only one facility reported involving men in its FP program as supporters of wives accessing this service. The contraceptive uptake at this centre was good, even to the extent of men collecting pills for their wives. Health workers were working closely with men, educating them on the importance of FP. This created interest and motivated husbands to support their wives.

Health workers at the other health facilities lacked the capacity to work with men. They suggested the need for further training on counselling, gender issues and interpersonal communication skills.

### Literacy and reproductive health outcomes

The association between literacy - the stated ability to read and write - and issues pertaining to reproductive health was explored by bivariate analysis as outlined in Table 5.

**Table 5:** Bivariate analysis of reproductive health outcomes against literacy (defined as the ability to read and write)

**Outcomes**	**P Value**
Knowledge of antenatal care	0.004
Spousal discussion on antenatal care	<0.001
Wife attended ANC clinics	<0.001
Knowledge of supervised birth	0.002
Spousal discussion on supervised birth	<0.001
Wife delivered at a health facility	<0.001
Knowledge of family planning methods	<0.001
Spousal discussion on family planning	<0.001
Wife using FP methods	<0.001

Literacy was strongly associated with the respondents’ discussion of SRH issues with their wives, and there was a very strong relationship between the respondent’s literacy status and their wives’ attendance at antenatal clinic, the likelihood of a supervised birth and utilisation of family planning.

## Discussion

Illiteracy, inadequate knowledge, cultural factors and lack of appropriate services were found to have negatively influenced male participation in SRH. It was widely considered that pregnancy, child birth and rearing of children are solely the role of women. Priority for many men was given to social obligations in order to gain recognition and maintain their status in the community. Though men are not direct beneficiaries of safe motherhood services, their understanding and participation and support is crucial in order for women to access basic reproductive health services. Whilst many other factors contribute to SRH and Child Health indicators the low level of male involvement is a major determinant.

In many societies in PNG, including those in the study district, the patriarchal structured society, in which gender rights strongly favour men, predominates. Traditionally men have been and still are the principal decision makers in social, economic and political aspects of family and village life. In most societies gender determines the roles and responsibilities of men and women and these cultural factors were found to significantly influence men’s sexual and reproductive behaviour and attitudes in this study. Many men in our study considered safe motherhood to be women’s responsibility. Lauglo noted that understanding gender systems benefits from examining the relationships between men and women as well as their respective roles [19]. This is important when developing interventions where gender norms are a barrier but change is only likely to occur with improved education and with efforts to encourage male involvement in all aspects of reproductive health.

In the study area, as in many other parts of PNG, societal norms (taboos) prohibit males from assisting and witnessing births and some men indicated that the presence of male health workers at a delivery facility was a reason for their wives to deliver in the village. Health authorities have tried to ensure that every facility has female staff, but this has not always been possible in isolated areas.

Men’s literacy, even when loosely defined as self-reported ability to read and write - was strongly associated with their knowledge of SRH issues, discussion with their wives, and with their wives’ utilisation of SRH services. The wives of the respondents who were knowledgeable about ANC and supervised births were more likely to access these services than were those of many of the men who were unaware of their importance (p < 0.001). These findings were similar to studies from India [20] and Nepal [21] where men who received antenatal education provided better support to their wives, which led to increased ANC attendance and health facility delivery.

Although many men had heard about FP, only a small proportion of participants and their wives were using contraceptives. Many were unaware of the importance and benefits of family planning. Factors such as wanting more children and fear of religious condemnation and of partners having extra marital relationships hindered couples from accessing contraceptives. Many who would have benefited from the use of condoms did not access them due to misunderstanding of their dual functions as contraceptives and as.a means of disease prevention.

Vasectomy as a form of family completion was not known by the participants in the study and few knew of tubal ligation. Many expressed an interest in vasectomy and wanted more information on the method. Counselling and quality services associated with strong information and sensitization campaigns on vasectomy were proven to be successful in African countries [22] and vasectomy programmes have been well accepted in some areas of Papua New Guinea [23].

Men’s perspectives on FP in this study are similar to those reported from Nigeria where men allowed women to negotiate future FP contraceptives only after bearing the required number of children [24]. This could be due the misconception that female methods result in permanent sterilisation and family planning education should target such issues. In many rural areas, it is difficult to maintain contraceptive continuity, especially with short term methods. Methods such as IUDs and implants are more suitable for women in these rural areas, but are not widely available in PNG.

Spousal communication is important in family planning. Condoms, periodic abstinences and withdrawal require communication and negotiation between partners to be used effectively [25]. Our study found that men sometimes deliberately avoided discussions when wives tried to raise the issues. Absence of spousal discussion makes it difficult for women to negotiate contraceptive use, a common determinant discovered in many reproductive studies [22]. Spousal discussion is likely to increase with improved education of both males and females and with improved male involvement in SRH services.

Involving men, and obtaining their support and commitment to family planning, is of crucial importance. PNG cultural gender roles are similar to those in many African [22] and Indian [26,27] societies where men are responsible for decisions on fertility and contraceptive use. The husband’s support is a good predictor for future practice and continued use of FP methods [22].This suggests the need for couple centred family planning and reproductive programs. Janine reported from a study in Indonesia that couple centred approaches to FP are viable in patriarchal societies [28].

The risk of contracting HIV among women in a non-high risk group has been found to be largely determined by their male partner’s behaviour [29]. In PNG it is assumed that most transmission is from husbands to wives. Thus engaging men more extensively in HIV prevention is likely to reduce women’s risk of HIV infection [30]. Most participants were aware that HIV/AIDS is transmitted during sexual intercourse with an infected person and they were familiar with the prevention strategies of one sexual partner and avoiding prostitutes. Knowledge of prevention of HIV through the use of condoms was acknowledged by many but did not necessarily translate into safe sex behaviour. Half of those that had sexual relationships with non-cohabiting partners did not use condoms. Such behaviour poses risks for wives who, owing to cultural norms, are not able to negotiate safe sex.

Domestic violence may result when the HIV status of women is disclosed to husbands, especially when the husband is negative [31]. It is important for both partners to know their HIV and VDRL status so that appropriate care can be sought from service providers and so that steps to prevent Mother to Child Transmission can be taken. Unfortunately HIV and VDRL screening are not widely available outside of main centres of PNG.

Men’s understanding of HIV infection and their attitudes towards prevention are key factors in containing the disease and in preventing infection of their wives and children. Most study respondents have stable family lives but some occasionally exhibit high risk sexual behaviour. Many indicated that they would be afraid to disclose their HIV/AIDS status to their wives or others if they were found to be positive.

The understanding that HIV/AIDS is a deadly disease for which there is no cure had apparently prompted some men to change their sexual behaviour. They wanted to be faithful to their wives. However many of those in a polygamous marriage did not view this as a threat to HIV/STI transmission but as a positive solution to reducing the risks of contracting HIV/STI infections. A Kenyan study showed that behavioural change messages directed at women had a low potential for preventing STIs and HIV/AIDS [32]. Such programmes directed at men are more likely to impact their attitude and sexual behaviour.

The concept of male involvement in SRH is still largely underdeveloped in PNG. Services currently available for men at rural health facilities are inadequate. In most health facilities there is no involvement of men in Safe Motherhood programmes. Many health workers are uncomfortable working with male clients in SRH programs due to lack of appropriate knowledge and skills. Condoms are often not readily available and even if they are the fact that they are often distributed by female health workers is a barrier to access. The study found that although men were willing to participate in SRH activities the environment was not conducive to their involvement.

The association between men’s literacy and their wives‘SRH practices was striking. Whilst much focus is put on the importance of female education in improving health outcomes the education of males is also clearly important. Literacy enables access to information relevant to health. It is important that education on issues of sexual and reproductive health be included at all levels, beginning at primary school.

## Conclusion and recommendation

Inadequate knowledge, cultural factors and lack of appropriate services adversely affect men’s involvement in their wives’ and their own sexual and reproductive health. Implementing the following recommendations is likely to improve men’s involvement and consequently improve the sexual and reproductive health of their wives and their communities.

➢ The current female oriented awareness and education programmes on family planning and safe motherhood should be re-examined and adjusted to involve men.

➢ Strong advocacy training for program managers, service providers, teachers and male leaders is required in order to enable them to propagate SRH initiatives in their respective communities.

➢ Peer educators and community leaders should be trained and supported to work with men in their respective areas.

➢ Service providers should be trained and equipped with appropriate knowledge and skills to accommodate men in their sexual and reproductive health programmes.

➢ Male friendly clinics should be established at all facilities to address men’s sexual and reproductive health needs.

➢ Educational programs on SRH should be integrated into the existing curriculum for young people starting, at primary school level.

## Endnote

^a^There has been debate as to the accuracy of this figure but even if the more recent estimate of 250/100 000 (2008 adjusted estimate from Unicef State of the World’s Children 2012) is more accurate it is still alarmingly high.

## Competing interests

The authors declare that they have no competing interests.

## Authors’ contributions

SK designed, organised and conducted the study, collated and made the initial analysis of the data, and wrote up the research project as a Master’s thesis. JV assisted in drafting the manuscript from the thesis and with some data analysis. PCC supervised the development of the project and the thesis and advised during the preparation of the manuscript. All authors read and approved the final manuscript.

## References

[B1] UNFPA International Conference on population and development-ICPD- program of action a/CONF.171/13/Rev.1 1995 http://www.unfpa.org/public/home/sitemap/icpd/International-Conference-on-Population -and- Development (cited on 13/09/2013)

[B2] Annual Health Sector Review Assessment of sector performance 2006–2010 2011 Report: National

[B3] Demographic and Health Survey (DHS) Papua New Guinea National Report 2009 Port Moresby: National Statistical Office

[B4] UNAIDS Papua New Guinea releases new HIV prevalence estimates from the National Department of Health and the National AIDS Council Secretariat 2010 http://unaids.org/en/resources/presscentre/featurestories/2010/august/20100826fspng/ (Cited on 03/06/2013)

[B5] STI, HIV/AIDS Surveillance Unit The 2009 STI, HIV and AIDS Annual Surveillance Reports 2009 Port Moresby: The Papua New Guinea National Department of Health

[B6] Padian N Shiboski S Glass S Vittinghoff E Heterosexual transmission of Human Immunodeficiency Virus (HIV) in Northern California: results form a ten year study Am J Epidemiol 1997 146 4 350 357 10.1093/oxfordjournals.aje.a009276 9270414

[B7] European Study group on Heterosexual Transmission of HIV Comparison of female to male and male to female transmission of HIV in 563 stable couples BMJ 1992 304 6830 809 813 1392708 10.1136/bmj.304.6830.809 PMC1881672

[B8] Ditekemena J Koole O Engmann C Matendo R Tshefu A Ryder R Colebunder R Determinants of male involvement in maternal and child health services in sub-Saharan Africa: a review Reproductive Health 2012 9 32 doi:10.1186/1742-4755-9-32 10.1186/1742-4755-9-32 23171709 PMC3573948

[B9] Byamugisha R Astrom AN Ndeezi G Karamagi CA Tylleskar T Tumwine JK Male partner antenatal attendance and HIV testing in eastern Uganda: a randomized facility based intervention trial J Int AIDS Soc 2011 14 1 43 doi:10.1186/1758-2652-14-43 10.1186/1758-2652-14-43 21914207 PMC3192699

[B10] Mbizvo MT Bassett MT Reproductive health and AIDS prevention in sub-Saharan Africa: the case for increased male participation Health Policy Plan 2006 11 1 84 92 10155880 10.1093/heapol/11.1.84

[B11] Pachauri S Male involvement in reproductive health care. South and East Asia, Population Council, New Delhi J Indian Med Assoc 2001 99 3 138 141 11478756

[B12] Ezeh AC The influence of spouses over each other’s contraceptive attitudes in Ghana Stud Fam Plann 1993 24 3 163 174 10.2307/2939231 8351697

[B13] Joesoef MR Andrew LB Budi U Husband’s approval of contraceptive use in metropolitan Indonesia: program implications.’ Stud Fam Plann 1988 19 3 162 168 10.2307/1966751 3406964

[B14] Nzioka C Programming for male involvement in reproductive health; Research on men and its implications on policy and programme development in reproductive health: report of the meeting of WHO Regional Advisors in Reproductive Health 2001 Washington DC, USA whqlibdoc.who.int/hq/2002/WHO_FCH_RHR_02.3.pdf (Cited on 03/06/2013)

[B15] Collumbien M Hawkes S Missing men’s message; does the reproductive health approach respond to men’s health needs? Culture, Health and Sexuality 2000 5 7 135 150 10.1080/136910500300769 12295879

[B16] Polit DF Hungler BP Nursing research and principles 1999 6 Williams and Wilkins: Lippincott

[B17] Parahoo K Nursing research, principles. Process and issues 2006 2 Palgrave Macmillan Limited

[B18] Strauss A Corbin J Basics of qualitative research; techniques and procedures for developing grounded theory 1998 2 New Delhi: SAGE Publications, Thousand Oaks, London

[B19] Lauglo M Gender, reproductive health and reproductive rights. World Bank institute, paper prepared for adapting to change core course 1999 http://www.info.worldbank.org/etools/docs/library/48447/m1s5lauglo.pdf (cited on 12/09/13)

[B20] Singh A Ram F Male involvement during pregnancy and child birth: evidence from rural ahmadnagao, India Population Review 2009 48 1 83 102 010.1353/prv.0.0016

[B21] Mullany BC Becker S Hindin MJ The Impact of including husbands in antenatal health education services on maternal health practices in urban Nepal: results from a randomized controlled trial Med Health Edu Res 2007 22 2 166 176 doi:10.1093/her/cy1060. 10.1093/her/cyl060 16855015

[B22] Toure L Male involvement in family planning a review of selected program initiatives in Africa 1996 http://pdf.usaid.gov/pdf_docs/PNABY584.pdf (Cited on 28/05/2013)

[B23] O’Connor M Non-Scalpel vasectomy in PNG: an evaluation, current issues and recommendations regarding going to scale 2007 Port Moresby: The Papua New Guinea National Department of Health

[B24] Bankole A Desired fertility and fertility behavior among the Yoruba of Nigeria: a study of couple preferences and subsequent fertility Popul Stud 1995 49 2 317 328 (12)10.1080/0032472031000148536 10.1080/0032472031000148536

[B25] Greene EM Mehta M Julie P Deirdre W Bankole A Singh S Involving Men in reproductive health; contributions to development 2009 http://www.unmillenniumproject.org/documents/Greene_et_al-final.pdf. (Cited on 11/04/2011)23802102

[B26] Saha KB Singh N Chatterjee Saha U Roy J Male involvement in reproductive health among the scheduled tribe: experienced from khairwars of central India Rural Remote Health 2007 7 2 605 17511523

[B27] Sharma A Men involvement in reproductive health: women’s perspective Family Welfare 2003 49 1 1 9

[B28] Janine L Barden OF Ilene S Speizer Indonesian Couples’ pregnancy ambivalence and contraceptive use Int Perspect Sex Reprod Health 2010 36 1 36 43 10.1363/3603610 20403804 PMC3883040

[B29] Hunter DJ Maggwa BN Mati JK Tukei PM Mbugua S Sexual behaviour, sexually transmitted diseases, male circumcision and risk of HIV infection among women in Nairobi, Kenya AIDS 1994 8 1 93 99 10.1097/00002030-199401000-00014 8011242

[B30] Mudota D Men's Sexual and reproductive health rights in the context of HIV Three-2010 http://www.kit.nl/net/KIT_Publicaties_output/showfile.aspx?e=1709 (cited on 12/09/13)24032114

[B31] Anyadike O The downside of male involvement in PMTCT, KENYA;HIV diagnosis can lead to domestic violence 2012 http://www.irinnews.org/printreport.aspx?reportid=94652 (cited on 12/09/13)

[B32] Daly CC Maggwa N Mati JK Risk factors for gonorrhoea, syphilis, and trichomonas infections among women attending family planning clinics in Nairobi, Kenya Genitourin Med 1994 70 3 155 161 8039777 10.1136/sti.70.3.155 PMC1195222

